# Metformin improves memory via AMPK/mTOR-dependent route in a rat model of Alzheimer’s disease

**DOI:** 10.22038/IJBMS.2023.73075.15879

**Published:** 2024

**Authors:** Reza Ale Mahmoud Mehraban, Parvin Babaei, Kambiz Rohampour, Adele Jafari, Zoleikha Golipoor

**Affiliations:** 1 Cellular and Molecular Research Center, School of Medicine, Guilan University of Medical Sciences, Rasht, Iran; 2 Department of Physiology, School of Medicine, Guilan University of Medical Sciences, Rasht, Iran; 3 Neuroscience Research Center, School of Medicine, Guilan University of Medical Sciences, Rasht, Iran

**Keywords:** Alzheimer disease, AMP-activated protein – kinase, Memory, Metformin, mTOR

## Abstract

**Objective(s):**

Metformin, as an insulin sensitizer, is a familiar antidiabetic drug. Increasing evidence points to metformin’s protective effects against Alzheimer’s disease (AD). However, the mechanism is not well understood. The present study evaluated whether inhibiting AMPK and activating mTOR could stop metformin from improving memory in rats with streptozotocin (STZ) -induced Alzheimer’s disease.

**Materials and Methods:**

Twelve-week-old Wistar rats, were injected 3 mg/kg STZ intracerebroventricularly on days 1 and 3 to develop the animal model. Metformin was applied orally at 100 mg/kg (17 days). Forty-five min before the retrieval phase, dorsomorphin (DM; AMPK inhibitor, 2 M) and MHY (mTOR activator, 0.1 M) were administered. Morris Water Maze (MWM) and shuttle box were utilized to measure spatial and passive avoidance memory, respectively. Congo red staining was used to identify cortical amyloid deposition.

**Results:**

The findings exhibited a considerable enhancement in spatial learning and memory in the metformin treatment group (*P*≤0.05). Injection of DM and MHY alone could not significantly change MWM and passive avoidance. Additionally, co-administration of DM and MHY increased escape latency (*P*≤0.001) and reduced the total time spent in the target quadrant (TTS) (*P*≤0.05) compared to the STZ+MET group during retrieval of MWM. Also, co-injection of DM and MHY increased step-through latency (STL) and decreased time spent in the dark compartment (TDC) compared to the STZ+MET group (*P*≤0.001).

**Conclusion:**

Metformin appears to have a therapeutic impact by activating AMPK and inactivating mTOR. As a result, it could be used as an Alzheimer’s treatment strategy.

## Introduction

Alzheimer’s disease (AD) is a progressive neurodegenerative disease. It is clinically identified by the gradual deterioration of memory and cognition. Alzheimer’s pathology usually includes amyloid plaques, neurofibrillary tangles, inflammation, oxidative stress, synapse destruction, and neuronal death (1). It is suggested that AD occurs mainly by binding amyloid plaques to insulin cell receptors, inhibiting intracellular insulin signaling, causing insulin resistance, and ultimately altering glucose metabolism and amyloid clearance (2). 

Since insulin signaling is essential in learning and memory, increasing insulin sensitivity can effectively combat dementia (3). Metformin, a well-known antidiabetic drug, is an insulin sensitizer. Crossing the blood-brain barrier affects the central nervous system. It helps improve insulin signaling pathway flow. Experiments have shown that in dementia models, metformin affects Aβ formation, reduces apoptosis, and enhances memory (4-7).

AMP-activated protein kinase (AMPK) is a crucial target of metformin. AMPK acts as an energy receptor and participates in many physiological processes like cell growth, metabolism, and apoptosis. Besides, it is a crucial factor affecting inflammation and insulin resistance associated with Alzheimer’s pathology (8, 9). Activating AMPK increases insulin sensitivity and inhibits hepatic gluconeogenesis, improving hyperglycemia. Besides, it regulates tau phosphorylation, amyloid-beta (Aβ) production, and autophagy, which are crucial in AD pathogenesis (10). Impairment of the AMPK signaling pathway is associated with disorders observed in AD (11). 

Metformin can inactivate mTOR and decrease mTOR signaling, reducing age-related pathologies, including movement disorders and insulin insensitivity. Decreased mTOR signaling may also benefit age-related diseases, such as AD (12, 13).  A chronic increase in mTOR activity may lead to the development of tau-related disorders (14). Besides, AMPK acts through the inhibition of mTOR, which modulates cellular metabolism, insulin secretion, insulin resistance, and autophagy (15).

There are few animal studies investigating the impact of metformin on memory disablement, and there are contradictions in their findings. For instance, Kuhla *et al*. reported that metformin elevated tauopathy and neurodegeneration in ApoE animals (16). The results of another study, however, indicate that metformin can prevent the decay of memory caused by hypobaric hypoxia with novel object recognition memory and Morris Water Maze (MWM) tasks (17).

In the current study, we first examined whether metformin improves memory damage in the STZ-induced rat model. Due to the interaction between metformin, AMPK, and mTOR, dorsomorphin as a selective inhibitor of AMPK and MHY as an activator of mTOR were applied in rats separately or in combination to understand the potential mechanisms of metformin.

## Materials and Methods

***Animals***

The study used 64 male Wistar rats aged three months and weighing 200–250 g. During a 12-hour light/dark period (lit 7:00 hr), Rats were housed in cages with standard laboratory chow and tap water, with unlimited access to food. Room temperatures were maintained at 22±2 °C. All animal experiments followed the guidelines of the “Institutional Guide for the Care and Use of Laboratory Animals.” Further, our experiments were accepted by the local ethics committee (code No: IR.GUMS.REC.1399.230).

The rats were assigned at random into eight groups (n=8), namely; 1) Saline, 2) STZ, 3) STZ+ MET, 4) STZ+ MET + DM, 5) STZ + DM, 6) STZ+ MET + MHY, 7) STZ+ MHY, 8) STZ+ MET + DM+ MHY. 

***Surgery***

For five days, the animals were handled and acclimated to the laboratory. Following anesthesia with ketamine (100 mg/kg) and xylazine (5 mg/kg), they were put in a stereotaxic device (Stoelting, Chicago, IL, USA). Using the Paxinos and Watson atlas of the rat brain (18), they were bilaterally cannulized (22 gauge) based on ventricular coordinates: DV=3 mm from the cranial surface; AP=0.8 mm from the bregma; lateral=1.6 mm from the midline. The skull was then screwed into place, and cannulae were attached with dental cement. Finally, animals were placed in recovery cages on warm pads. Seven days after surgery, microinjections were performed using 27-gauge needles and 10 µl Hamilton syringes connected to a short polyethylene tubing. Following the injection procedure, the needles were left for another minute before slowly being withdrawn.

***Drugs and treatments***

Streptozotocin (STZ; 3 mg/kg, Sigma, USA) was dissolved in 0.9% sterile saline (19). Then it was administrated in a volume of 10 µl into the lateral cerebral ventricles on days 1 and 3 of the procedures. Saline was injected in the same volume bilaterally in the saline group. 

Dorsomorphin (DM; Selleckchem, USA), an inhibitor of AMPK, was dissolved in 0.9% sterile saline. MHY1485 (Selleckchem, USA) is an activator of mTOR dissolved in saline and 5% dimethyl sulfoxide (DMSO). Animals received either combined or solitary dose of DM (10 µl, at a dose of 2 µM, ICV) or MHY (10 µl, at a dose of 0.1 μM, ICV) 45 min before the retrieval phase of behavioral tests (19).

**Figure 1:**
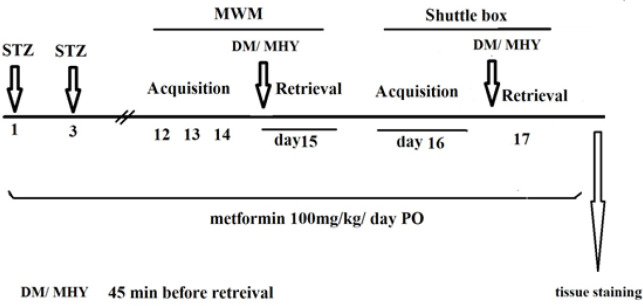
Experiment design showing the time course of drug administration and behavioral tests in rats in different groups

A daily dose of 100 mg/kg of metformin was administered orally from day 1 to day 17 of the study (20). The experimental process for behavioral tests and treatments is shown in Figure 1. 

***Behavioral test***

*Morris water maze*

 The MWM was applied to assess spatial memory. The maze consists of a dark pool (148 cm diameter, 60 cm height) that contains water and a rectangular stand (10 cm) attached to a target quadrant, 1.5 cm below the water level. The temperature of the water was kept at 26±2 °C. The evaluation was performed during four days (3 days of acquisition, one day of test), on days 12 to 15 for each group.  There were four blocks for the first three days, each consisting of 4 trials, each lasting 90 sec with an interval of 30 min. On day 4, the probe test was performed for 90 sec. Independently, the animals found the hidden stand by performing the MWM task. The animal’s behavior was traced by a camera connected to the autovision system (ethovision 12, Noldus Inc., the Netherlands), which allows for measuring swimming speed, the time spent to discover the stand (escape latency), and the total time spent in the target quadrant (21).

*Passive avoidance task*

The passive avoidance appliance comprised a two-chamber dark/light shuttle box (20×20×30 cm high) with a guillotine door (7×9 cm) separating the chambers. In both parts, stainless steel bars (2 mm diameter) were separated 1 cm apart to form the bottom. The black room’s floor could be electrified. A soundproofed room was used to house the appliance. Passive avoidance using the step-through method was used to find the memory (19). 

 For habituation, rats were put in the lighted room and were permitted to go voluntarily inside both sides for 5 min. This action was repeated once more after 30 min. On the teaching day, rats were put into the light part; after 10 sec, the gate was uplifted, and the crossover latency was marked. After the rat entered the black side, the door was closed at the back of it, and an electric shock (1 mA, 50 Hz, 3 sec) was applied to the grid. Retention tests were done 24 hr after teaching to evaluate memory. 

The animals were placed in the light room, and after 10 sec, the gate was unlocked, and the step-through latency (STL) and the time spent in the dark compartment (TDC) were measured up to 300 sec. Animals did not receive any shock from the grid floor during the session.

*Congo red staining*

The Congo red stain was used to look for the cortex’s Aβ plaques. Congo’s red color forms non-polar hydrogen bonds with amyloid plaques. After behavioral testing, rats received 250 ml of 4% paraformaldehyde and then 0.01 mol/l phosphate-buffered saline (PBS). All tissues were cut up and fixed in 4% paraformaldehyde for 48 hr before being dehydrated in 20% and 30% sucrose, respectively. The sections were diced using a cryostat microtome at –20 °C. Congo red solution was immersed in tissue sections for 18 min, 20 min of running tap water, and 10 sec of weak alkali. The tissues were then incubated in hematoxylin for 5 min before being washed in water for 30 min and going through the same dehydration, clearing, and coverslipping steps in the way that is done in H&E staining. A light microscope was used to observe the amyloid-beta deposition (22).

***Statistical analysis***

Prism software version 9 was used to analyze the data. The normality of the data was determined using Shapiro-Wilk. One-way analysis of variance (ANOVA) was used to compare the results with a normal distribution, followed by the Tukey test, and a two-way test to compare the data with the Morris test. *P*<0.05 was considered significant, and data were reported as Mean±SEM.

## Results

***Effects of metformin, dorsomorphine, and MHY on spatial memory deficit***

**Figure 2:**
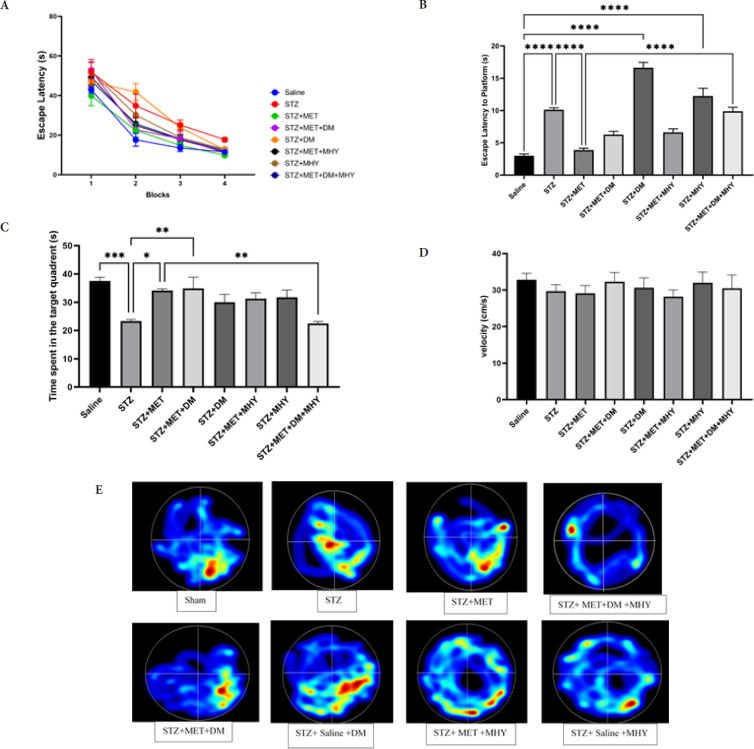
Effect of STZ, metformin, DM, MHY, and DM+MHY on MWM in rat in different groups

There were measurements of the wait time to find the escape platform, the total time spent in the target quadrant (TTS), the swim speed, and the escape latency in the probe. During acquisition trials, the reduction in escape latency indicated that all rats successfully learned to discover the sheltered stand in MWM (ANOVA Two-way, *P*<0.0001, Figure 2A). 

Besides, there was diversity in escape latency and TTS in probe tests between groups. As demonstrated in Figure 2B, a significant increase in escape latency was observed in the STZ group contrary to the saline group (*P*=0.0001), but a significant reduction was observed in STZ+MET against the STZ group (*P*=0.0001). Also, escape latency was elevated in the STZ+MET+DM+MHY group contrary to STZ+MET(*P*=0.0001).

In addition, TTS was significantly reduced in the STZ group (*P*=0.006) against the saline and increased in the STZ+MET+DM group (*P*=0.018), contrary to the STZ group. Also, TTS decreased in the STZ+MET+DM+MHY group compared to the STZ+MET group (*P*=0.007; Figure 2C, E). Our data did not show a noticeable change in swimming speed between the groups (Figure 2D). Therefore, latency time in the acquisition, probe, and TTS was applied as memory indexes but not mobility.

***Effects of metformin, dorsomorphine, and MHY on passive avoidance memory deficit***

**Figure 3:**
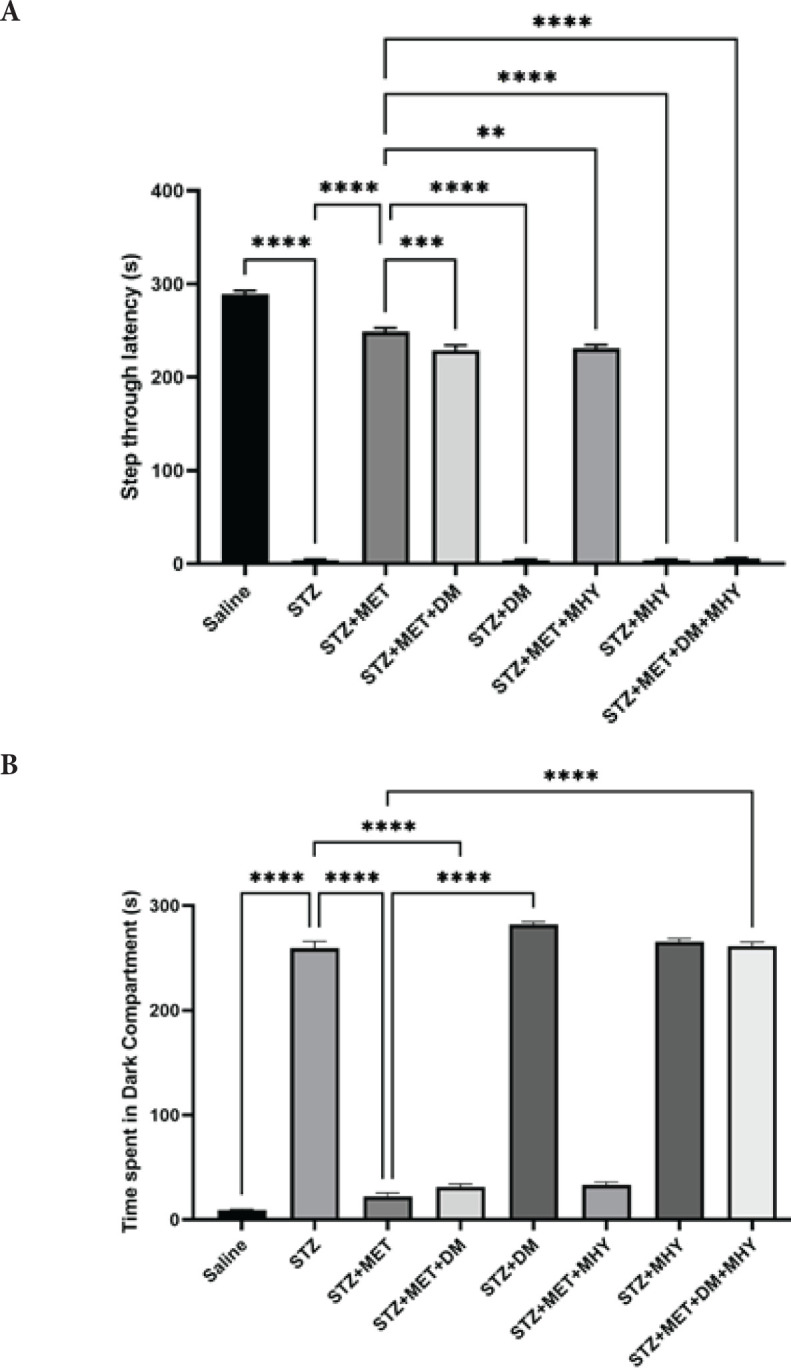
Effect of STZ, metformin, DM, MHY, and DM+MHY in rats in different groups

The results of passive avoidance memory in retrieval tests are shown in Figure 3. There were significant differences between groups in STL and TDC analyzed by one-way ANOVA. 

STZ group exhibited a shorter STL (*P*=0.0001) and longer TDC (*P*=0.0001) than the sham. STL was decreased in STZ+MET+DM (*P*=0.009), STZ+MET+MHY (*P*=0.004), and STZ+MET+DM+MHY (*P*=0.0001) compared to the STZ +MET group. In addition, TDC was increased in STZ+DM (*P*=0.0001) and STZ+MET+DM+MHY(*P*=0.0001) compared to STZ+MET. Based on these results, combined therapy appears to improve the recall of avoidance memories. 

***Effects of metformin, dorsomorphine, and MHY on Aβ plaque deposits***

**Figure 4:**
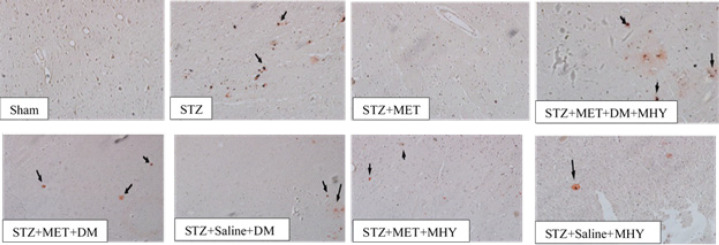
Effect of STZ, metformin, DM, and DM+MHY on Cogno red stained cortex tissue in rats in different groups

Staining with Congo red was applied to detect the Aβ plaque deposition. As illustrated in Figure 4, all groups except saline and STZ+MET showed positive Congo red staining which surrounds shrunken neurons with irregular morphology. 

The injection was performed 45 min before retrieval

A) escape latency time in 4 blocks in the acquisition phase of spatial memory. (ANOVA Two-way; *P*<0.0001). B) escape latency time in the probe test of spatial memory in MWM (ANOVA one way, Tukey post hoc). C) total time spent in the target quadrant in probe test of spatial memory in MWM. D) Comparison of swimming speed (velocity) in MWM among groups. E) heat maps showing the representative MWM tracking of groups during retrieval trials, increasing color intensity (arbitrary scale) represents increased time spent. Data are presented as mean±SEM. * *P*<0.05, **P<0.01, ***P<0.001, ****P<0.0001 (n=8).

STZ: Streptozotocin; DM: Dorsomorphin; MWM: Morris water maze

A) Alterations in step-through latency and B) time spent in the dark compartment. Data are presented as mean±SEM. **P<0.01, ***P<0.001, ****P<0.0001, (ANOVA one way, Tukey* post hoc*, n=8).

STZ: Streptozotocin; DM: Dorsomorphin; MWM: Morris water maze

Light micrographs of Aβ plaques. The black arrow shows Aβ plaques (Congo red staining, original magnification×100).

STZ: Streptozotocin; DM: Dorsomorphin; MWM: Morris water maze

## Discussion

Based on the results of the current study, metformin can improve spatial and passive avoidance memory in rats with STZ-induced AD, confirming previous studies (23, 24). In addition, consistent with other studies (2, 22), injection of icv-STZ induced amyloid plaque formation while metformin treatment decreased cortical amyloid plaques. Farr *et al*. prescribed a daily dose of 20 mg/kg metformin for eight weeks in the SAMP8 mice model of spontaneous onset AD. They showed that metformin enhanced learning and memory by reducing APPc99 and phosphorylated Tau (23). Also, Ou *et al*. denoted that metformin (200 mg/kg) attenuated spatial memory deficit and hippocampal neuronal loss in APP/PS1 mice. Besides, a reduction of Aβ plaque in the hippocampus was observed (25).

Conversely, Kuhla *et al*. reported that the 18-week treatment with metformin aggravated the neurodegenerative process in the ApoE-/-mice model. Increased tau phosphorylation and decreased Neu-N-and PSD95-positive cells were observed (16). Variables such as dose, duration of treatment, and type of dementia influence different reported results. For instance, in one study, lower doses of metformin (100mg/kg, 14 days) improved scopolamine-induced dysfunction in both MWM and passive avoidance tests, which were associated with a significant reduction of inflammatory markers such as TNFα and total Akt compared to the rivastigmine treatment group. Akt is a cellular kinase known as a significant regulator of tau. Metformin showed a protective effect at the average human dose used to treat diabetes (20).

AMPK inhibitor (DM) and mTOR activator (MHY) were used to demonstrate the mechanism underlying the protective effect of metformin. The findings demonstrated that although DM and MHY administration alone could not cause significant alteration, co-injection of these two agents had a remarkable impact on spatial and avoidance memory and amyloid plaques in the rat. The AMPK/mTOR signaling pathway was considered in several studies (26, 27), though the results are contradictory. For instance, Kickstein *et al*. emphasized that AMPK activation alone is insufficient to mimic the action of metformin on tau phosphorylation, which is very important in the formation of neurofibrillary tangles in Alzheimer’s patients. They demonstrated that metformin inhibited mTORC1 and activated PP2A, and this effect of metformin was independent of AMPK (28). On the other hand, researchers stated that the neuroprotective influence of metformin is AMPK-dependent. They reported that AMPK-activated metformin could protect human neural stem cells against Aβ-induced mitochondrial dysfunction (29). It is well established that metformin exerts its effect partly by stimulating AMPK. A recent report showed that metformin via activating AMPK effectively decreased both Aβ deposition and the soluble form of Aβ, while Compound C (AMPK inhibitor) increased Aβ burden. The APP/PS1 mice demonstrated a significant rise in phosphorylated AMPK expression and a significant reduction in mTOR expression and Bace1, indicating that the alteration of these proteins is likely to be related to the neuroprotection conferred by metformin. (25). 

mTOR is known as a mechanistic target of AMPK (30). The previous study found that metformin activates AMPK, which inhibits mTOR activity via an impact on its downstream target, p70S6 kinase (S6k1)(31). S1K1 is an essential effector of mTOR signaling, which adjusts the APP process by influencing β- and γ-secretases. Besides, it is highlighted that mTOR activation promotes Aβ production and deposition (26). Consistent with our observations, it is suggested that the effect of metformin in preventing amyloid plaque deposition is due to its impact on increasing AMPK and decreasing mTOR activity (25).

In addition to regulating metabolic and cellular processes, metformin provides energy to the body. Metformin directly correlates to improved cognitive performance, according to growing data. More importantly, it was found that metformin is safe and well-tolerated. So, determining the mechanism of action of this medicine in preventing cognitive dysfunction is critical. (32). Our data provide evidence that each of the two AMPK and mTOR pathways alone can mediate the action of metformin on memory improvement. However, the involvement of both pathways simultaneously will be more effective. The present study’s findings suggested that metformin improved memory by activating both signaling pathways in parallel.

## Conclusion

To summarize, the current investigation identifies the benefits of metformin for treating memory impairment based on its clinical impact. The findings showed that metformin improved spatial and passive avoidance memory and the Aβ plaques in rats with STZ-induced Alzheimer’s disease.

## Authors’ Contributions

A J and K R designed the study and prepared the manuscript. P B supervised the MWM task. R M performed the behavioral experiments. Z G performed the tissue staining. A J analyzed and interpreted the data. All authors read and approved the final manuscript. All authors have accepted responsibility for the entire content of this manuscript and approved its submission.

## Ethical Approval

This study has been approved by the ethics committee of Guilan University of Medical Sciences (code No: IR.GUMS.REC.1399.230).

## Conflicts of Interest

The authors declare no conflicts of interest.
